# Analysis and validation of diagnostic biomarkers and immune cell infiltration characteristics in osteoarthritis by integrating bioinformatics and machine learning

**DOI:** 10.3389/fimmu.2025.1596912

**Published:** 2025-08-20

**Authors:** Tianyang Li, Jinpeng Wei, Hua Wu, Chen Chen

**Affiliations:** Department of Orthopedics, Third Hospital of Shanxi Medical University, Shanxi Bethune Hospital, Shanxi Academy of Medical Sciences, Tongji Shanxi Hospital, Taiyuan, China

**Keywords:** osteoarthritis, exosome, machine learning, immune infiltration, biomarkers

## Abstract

**Objective:**

Exosomes as important carriers of intercellular communication have frequently appeared in recent studies related to osteoarthritis (OA), while the specific mechanism of exosome action in osteoarthritis remains unclear. The aim of this study was to identify potential exosome-related biomarkers in osteoarthritis, to explore the role and mechanism of exosome-related genes in articular cartilage.

**Methods:**

The data on exosome related genes and normal and OA cartilage genes were obtained through online databases. The potential mechanisms of these genes were revealed by multiple gene enrichment analysis algorithms. Machine learning methods were utilized to identify exosome-related differential genes (ERDEGs) with highly correlated OA features (Hub OA-ERDEGs). In addition, we created a nomogram to assess the ability of Hub OA-ERDEGs to diagnose OA. Single-sample gene set enrichment analysis (ssGSEA) was used to observe the infiltration characteristics of immune cells in OA and their relationship with Hub OA-ERDEGs.

**Results:**

The results of screening Hub OA-ERDEGs using machine learning algorithms show that: TOLLIP, ALB, HP, RHOBTB3, GSTM2, S100A8 and AKR1B1 were significantly up-regulated or down-regulated in OA samples and verified by qRT- PCR for validation. Using the ssGSEA algorithm, we discovered that 8 types of immune cell infiltration and 5 types of immune cell activation.

## Introduction

Osteoarthritis (OA) is one of the most common musculoskeletal diseases, and the global prevalence of OA is increasing year by year, and the age of onset of the disease is showing a trend of younger people, according to statistics, more than 22% of adults over the age of 40 years suffer from knee OA (1). OA is characterized by degeneration of articular cartilage, accompanied by subchondral bone remodeling, synovial inflammation, and osteophyte formation, leading to joint pain, stiffness, and limited motion (2, 3). Despite the exciting advances we have made in the pathophysiology of OA, various challenges remain in its diagnosis and early prevention. Current diagnostic methods rely heavily on clinical functional assessment and imaging techniques, while these methods do not allow for the early diagnosis of OA and may also lead to diagnostic discrepancies due to subjective judgments (4). Therefore, a pressing need exists for innovative diagnostic markers and diagnostic strategies, which are important for the prevention and disease management of OA.

Exosomes are small extracellular vesicles, usually between 30 and 150 nanometers in diameter, secreted extracellularly by a variety of cell types. They facilitate intercellular communication by transferring proteins, lipids, and RNA between cells (5, 6). Exosomes from different cellular sources may act on articular chondrocytes through multiple pathways; exosomes from mesenchymal stem cells (MSCs) can activate the Nrf2 signaling pathway through TGFB1, a key gene, to promote cartilage regeneration and inhibit the formation of neutrophil extracellular traps (NETs) (7). Other studies have shown that exosomes can activate the PI3K-AKT-mTOR pathway, promote macrophage polarization towards anti-inflammatory M2-type macrophages, and enhance the antioxidant capacity and survival of chondrocytes (8). Meanwhile, microRNA-26b-5p in M2 macrophage-derived exosomes could further protect articular cartilage by targeting Toll-like receptor 3 (TLR3) and Collagen type X alpha 1 (COL10A1) (9). In contrast, endocytosis of exosomes derived from inflammatory fibroblast-like synoviocytes (FLS) promotes macrophage polarization towards pro-inflammatory M1 macrophages, inducing synovitis and exacerbating the progression of an OA model after injection of exosomes into the joint cavity of mice (10). In conclusion, exosomes of various cellular origins play important roles in influencing inflammation genesis, chondrocyte activity, and cartilage degradation, and highlight the importance of exosome research in elucidating the complex cellular interactions involved in the pathogenesis of OA and provide promising avenues for identifying novel diagnostic and therapeutic targets.

Incorporating bioinformatics and machine learning into the study of OA and exosomes can significantly enhance our understanding of this complex disease. Machine learning techniques are able to analyze large and complex data sets generated from genomic, proteomic, and metabolomic studies, allowing researchers to uncover complex patterns and relationships that may have been overlooked by traditional analytical approaches (11). In addition, machine learning can facilitate disease modeling, allowing for personalized treatment plans based on individual patient characteristics (12). In this study, machine learning algorithms were used to screen characteristic genes of OA, and single-sample gene set enrichment analysis (ssGSEA) algorithm was used to study the immune infiltration of OA and its relationship with hub genes. It provides guidance for advancing OA research, guiding clinical applications, and ultimately improving patient survival quality through targeted therapy.

## Methods

### Data preprocessing

The gene expression profile microarrays (GSE113825、GSE117999、GSE169077、GSE178557) of normal and OA cartilage samples were downloaded from the Gene Expression Omnibus database (GEO, https://www.ncbi.nlm.nih.gov/geo/). The datasets were first transformed into gene symbols, followed by background correction and normalization of each dataset in R software using the “limma” package, and integration of the two cartilage datasets using the “sva” package to eliminate batch effects (13). A two-dimensional PCA clustering plot was used to visualize the differences before and after removing the batch effect from the sample. Genes associated with exosomes were obtained from GeneCards Version 5.23 (https://www.genecards.org/), and we searched for exosome related genes (ERGs) and selected genes with correlations greater than 2 to be used for subsequent analysis (14).

Expression matrices of all genes in normal and OA cartilage samples were extracted from the training set and differences were analyzed using the limma package, using a threshold |logFC| > 0.25; P-value < 0.05 to obtain differential genes (DEGs) (15). Subsequently, the “ggplot2” package in R Version 4.2.3 was used to visualize the differential genes. The “ggvenn” package was used to intersect the DEGs and ERGs in the training set for subsequent analysis, and the genes obtained were named exosome-related differential genes (ERDEGs).

### Constructing protein interaction networks

To assess gene interactions among DEGs, a protein-protein interaction (PPI) network was constructed using the Interacting Genes Search Tool (STRING Version:12.0, https://cn.string-db.org/) database, with a confidence score of >0.7 as a cut-off criterion (16). Subsequently, the PPI network was visualized using Cytoscape software (version 3.8.2).

### Functional enrichment analysis

Gene Ontology (GO) is an organized knowledge base designed to provide standardized descriptions of genes and gene products. It mainly covers the annotations of Molecular Function (MF), Biological Process (BP), Cellular Component (CC) (17). The Kyoto Encyclopedia of Genes and Genomes (KEGG) integrates all kinds of biological information, especially data related to genomics and systems biology. The main feature is visualization using pathway maps to make complex biological processes easier to understand (18). Disease Ontology (DO) is a knowledge base for systematically describing and categorizing a variety of diseases and their associated characteristics that can enhance the effectiveness of disease research (19). To further understand the biological functions, signaling pathway enrichment and disease similarities of DEGs, we analyzed DEGs using the “clusterprofiler” package.

### Gene set enrichment analysis

Gene Set Enrichment Analysis (GSEA) is a bioinformatics method that is commonly used to analyze transcriptomic data to assess whether changes in gene set expression levels under different conditions are significant or not (20). This study utilized the GSEA package (v1.66.0) to conduct gene set enrichment analysis. The c2 (c2.cp.kegg_legacy.v2025.1.Hs.symbols.gmt) and c5 (c5.go.v2025.1.Hs.symbols.gmt) gene sets from the Molecular Signatures Database (MSigDB), along with the immune infiltration cell signature gene set defined by Yu et al (21), were applied to generate signature enrichment scores. To assess enrichment in OA cartilage samples, we used the “clusterprofiler” package to score GO and KEGG pathways and screened for the five pathways with the most significantly upregulated expression levels in OA.

### Machine learning algorithms screening hub characteristic genes

To further identify the characteristic genes, we used linear and nonlinear machine learning algorithms for the analysis. Linear models include Single-factor logistic regression (SFLR), which eliminates factors in the variables that are considered insignificant through the “glmnet” package, and the acquired gene expression data are used for the subsequent three nonlinear machine learning analyses (22). Least absolute shrinkage and selection operator (LASSO) logistic regression imposes L1 penalties on the regression coefficients by means of the “glmnet” package, which automatically selects significant features associated with the target variable (23). Support vector machine-recursive feature elimination (SVM-RFE) reduces the number of features efficiently by using the SVM function of the “e1071” package, reducing the computational cost and improving the interpretability of the model (24). Random Forest (RF) automatically evaluates the importance of features through the “random Forest” package, helping to identify the features that have the greatest impact on the target variable, facilitating feature selection and data analysis (25). Finally, using the “ggvenn” package, the intersection of the filtered feature genes of the three nonlinear machine learning models is defined as Hub OA-ERDEGs. The expression of Hub OA-ERDEGs in the training set and their location on the chromosome were subsequently visualized using the “ggpubr” and “circlize” package.

### Mouse chondrocyte culture and processing

Primary chondrocytes were isolated from the knee cartilage of four 5-dayold C57BL/6 male mice as follows: mice were disinfected by immersion in 75% alcohol. Under aseptic conditions, cartilaginous tissues of the knee joints of mice were dissected and isolated, and digested with 0.25% trypsin (Boster, China) at 37 degrees for 30 minutes. After centrifugation to remove trypsin, 0.2% collagenase type II (BioFroxx, Germany) was added and incubation was continued at 37 degrees for 4–6 hours. After centrifugation, the cells were resuspended in DMEM/F12 (HyClone, USA) medium containing 10% fetal bovine serum (FBS, Newzerum, New Zealand) and incubated at 5% CO2 and 37°C in an incubator. When the fusion rate reached 80-90%, the cells were digested with 0.25% trypsin and collected, and then transferred to 6-well plates at an appropriate density. After the chondrocytes were attached to the wall, the cells in the OA group were treated with 10 ng/mL concentration of IL-1β (R&D Systems, USA) for 24 hours, and the control group was only changed the culture medium. If not specified, first or second-generation chondrocytes were used for subsequent experiments. Animal studies are reported according to ARRIVE guidelines 2.0. Male C57BL/6 mice, 5-day-old, weighing approximately 3.5 g each, were purchased from Hubei Bainte Biotechnology Co., Ltd. All animal experiments were performed in accordance with a protocol approved by the Institutional Animal Care and Use Committee of Huazhong University of Science and Technology ( [2024] IACUC Number: 4741), and strictly followed the recommendations of the “Laboratory Animals of the National Institutes of Health”.

### Quantitative reverse transcription-PCR

Total RNA was extracted using the EZNA Total RNA kit (Omega Bio-Tek, USA) according to manufacturer’s instructions. Then we used 2 ug RNA to synthesize first strand cDNA with Reverse Transcription kit (Toyobo Life Science, Japan). PCR amplification was performed with SYBR Green Real-Time PCR Master Mix (Toyobo Life Science, Japan) on CFX96 (Bio-Rad Laboratories, USA) system. The relative gene expression level was calculated by comparative Ct method and normalized to internal control β-Actin. All PCR reactions were repeated in triplicates. Primer sequences for Hub OA-ERDEGs are shown in the **Supplementary Table S13**.

### Construction and evaluation of a nomogram

The nomogram will combine multiple variables of a predictive model in a simple visual way, providing an easy to understand and use method for assessing the risk or outcome of a patient-specific event. Therefore, we utilized the “rms” package to create a multivariate logistic regression-based outcome to generate a decision curve analysis (DCA) to assess the clinical utility of the nomogram (26). The “pROC” package was used to generate ROC curves to verify the reliability of the model, AUC > 0.75 indicates that this model has good performance (27).

### Immunological features of osteoarthritis

Single-sample gene set enrichment analysis (ssGSEA) is a method used to assess the enrichment of specific gene sets in a single sample. Unlike traditional gene set enrichment analysis, ssGSEA focuses on a single sample and is able to reveal biological features and pathway activities in the sample in greater detail (28). We calculated enrichment scores for normal and OA cartilage samples in immune cells using the “GSVA” and “GSEABase” packages, and used the “Ggpubr” package to plot box line plots, and the “reshape2” and “tidyverse” packages to plot heat maps of differential gene and immune cell correlations. Spearman correlation analysis was used to correlate Hub OA-ERDEGs with immune cells.

### Animal model

A destabilization of the medial meniscus (DMM) model was established in C57BL/6 mice aged six weeks. Mice were anesthetized with sodium pentobarbital before surgery. Following anesthesia, the knee joint area was prepared by shaving, disinfecting, and making an incision from the distal patellar to the proximal tibial region. The joint capsule was then opened, the patellar tendon retracted, and the medial meniscal ligament was exposed and transected. The Sham group underwent a similar incision procedure without transection of the medial meniscotibial ligament. Postoperative management included prophylactic penicillin administration and unrestricted ambulation in climate-controlled housing with ad libitum access to feed and water. At 12-week endpoint, bilateral joint specimens were harvested following humane euthanasia of three model group and three sham group animals.

### Histological evaluation

Knee tissues underwent post-fixation with 4% paraformaldehyde (Sigma-Aldrich) for 48-hour immobilization, followed by decalcification in 10% EDTA solution (Sigma-Aldrich). After complete demineralization, specimens were processed through paraffin embedding and sectioned sagittally at 6 μm thickness. Tissue sections underwent deparaffinization in xylene baths with subsequent graded ethanol rehydration. Histomorphological analysis was performed using H&E staining according to standardized protocols (Solarbio). For protein localization studies, immunohistochemical detection was implemented with specific antibodies targeting INOS (Abcam) and ARG1 (Abcam).

## Results

### Identification and PPI analysis of ERDEGs in osteoarthritis

**Figure 1:**
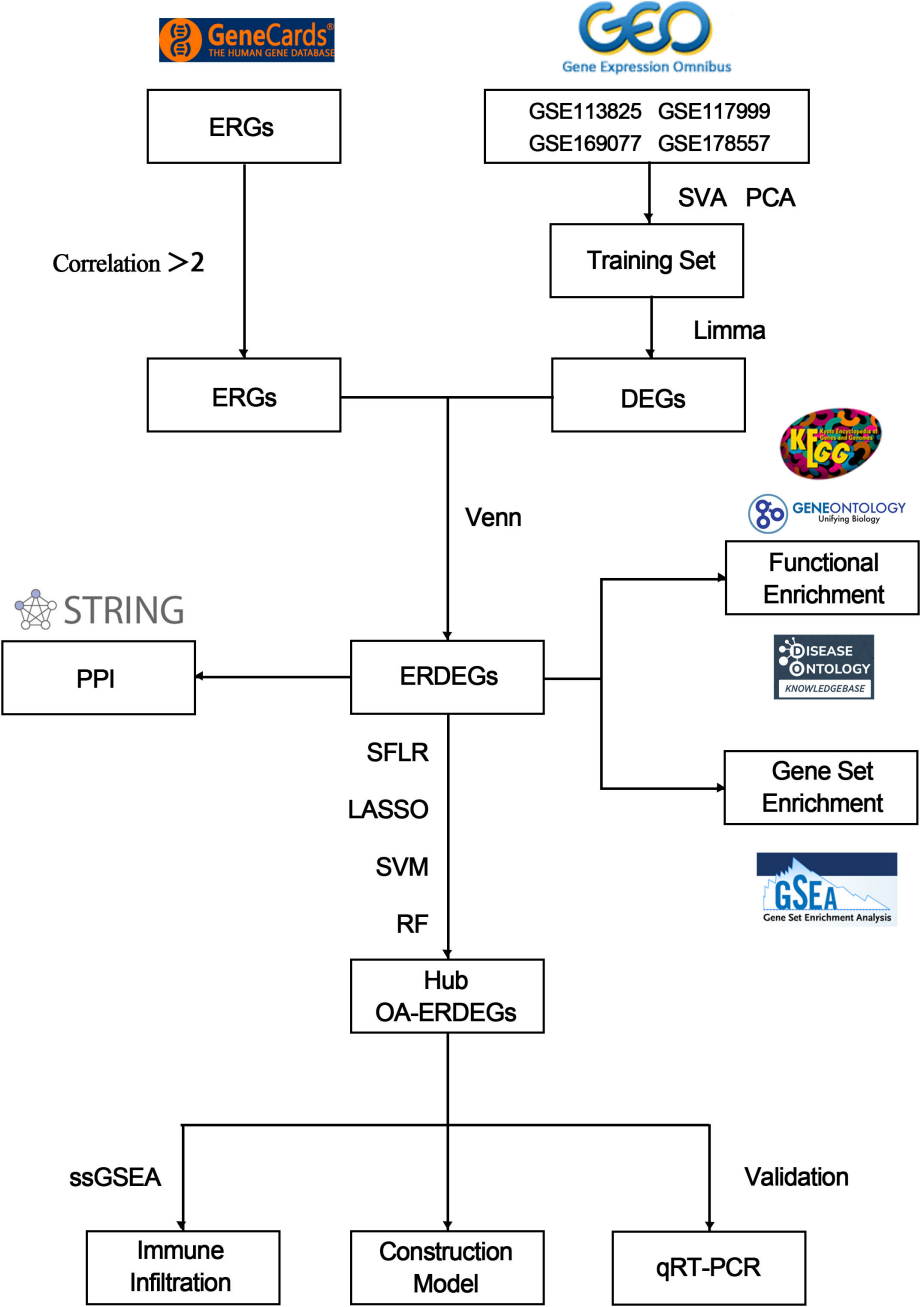
Flowchart for comprehensive analysis of exosome-related genes in OA.

**Figure 2:**
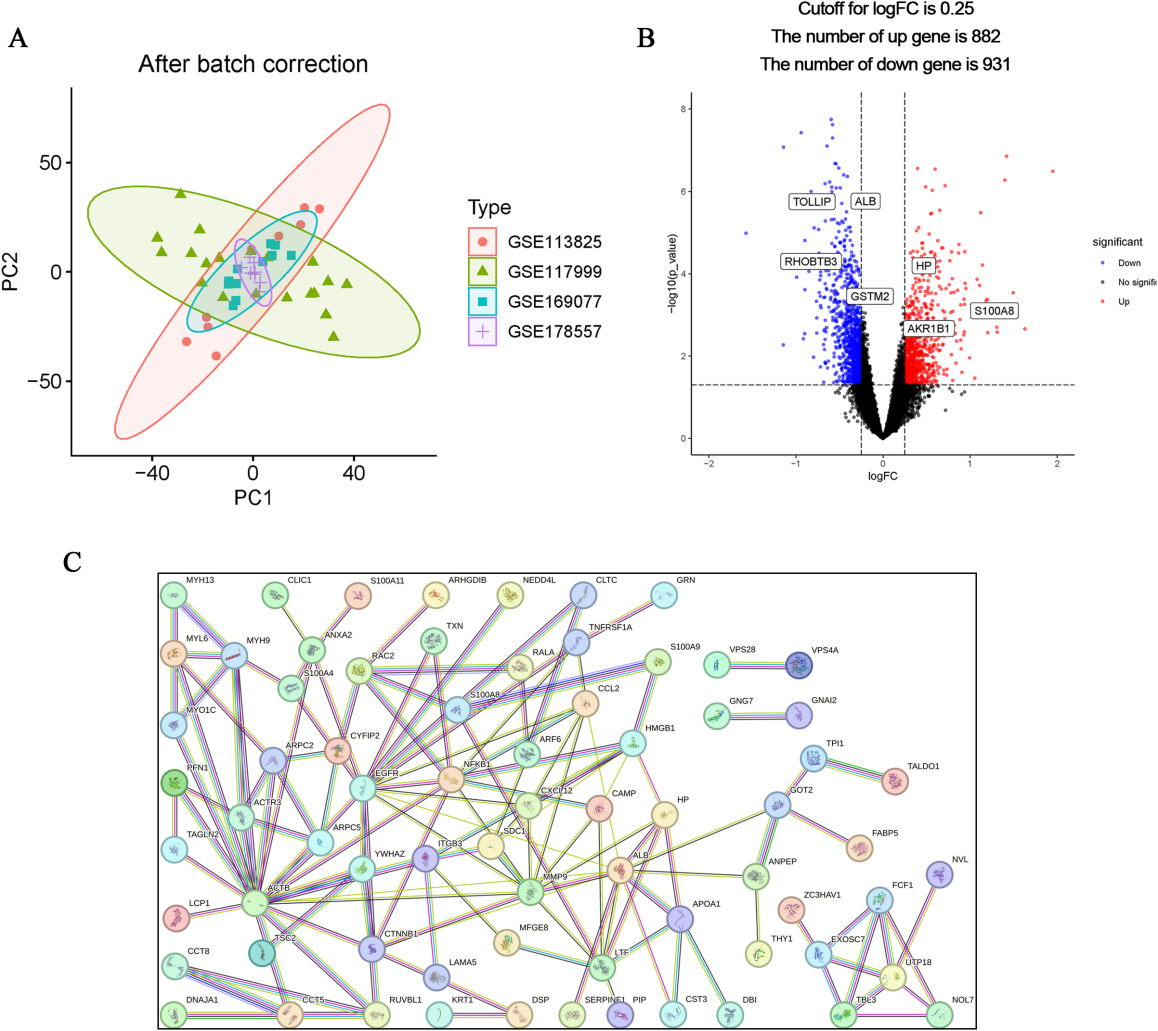
Identification and PPI analysis of ERDEGs. **(A)** Removal of batch effects and identification of DEGs in OA. **(B)** ERDEGs volcano plot. Red nodes indicate Deg upregulation, blue nodes indicate Deg downregulation, and gray nodes indicate genes with no significant differential expression. **(C)** Interaction map of 105 ERDEGs PPI protein network.

The flow chart of this study is shown in **Figure 1**. The calibrated dataset information is shown in **Supplementary Table S1**. The PCA results showed a significant difference between the samples from patients with OA and controls, and the batch effect was significantly removed, suggesting that the expression matrix was suitable for subsequent analyses (**Figure 2A**). 861 exosome-associated genes with correlations greater than 2 were obtained from the GeneCards database for subsequent analysis. (**Supplementary Table S2**). A total of 1813 DEGs in the dataset of OA-related genes were shown in the volcano map (**Figure 2B**). We subsequently intersected the DEGs with the ERGs by taking the intersection of the DEGs, and found 105 ERDEGs using the “VennDiagram” package, of which 73 genes were up-regulated and 32 genes were down-regulated in OA, and the **Supplementary Table S3** contains a detailed list of these related genes. PPI protein network Interaction analysis showed that ERDEGs interacted closely at the protein level (**Figure 2C**). The results of the PPI protein network interaction analysis are shown in **Supplementary Table S4**.

### Functional enrichment analysis of ERDEGs

**Figure 3:**
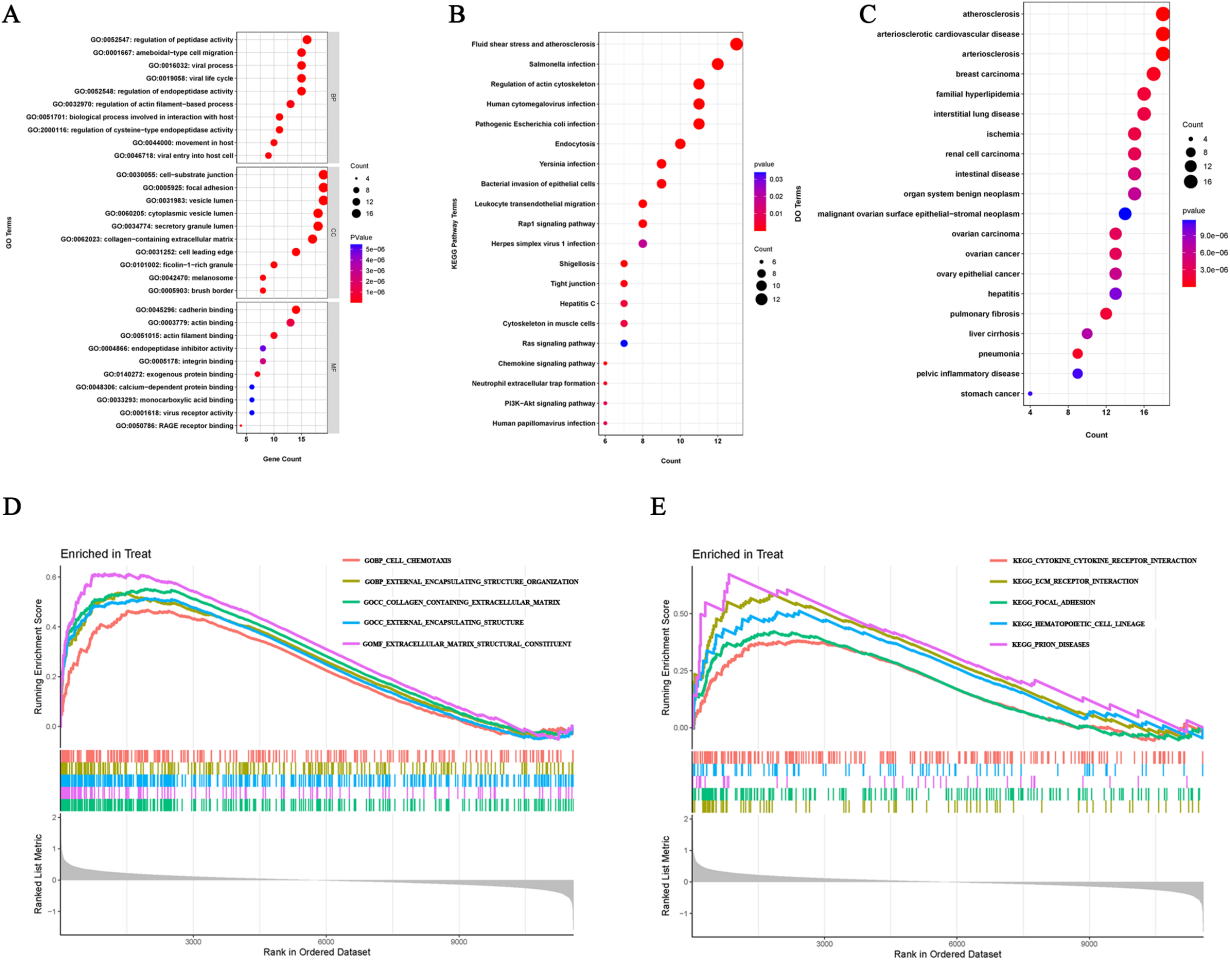
ERDEGs functional enrichment analysis. **(A)** GO enrichment analysis with BP, CC, and MF included. The bubble plots depict the ten most significantly enriched functions, where the size of the bubbles represents the number of DEGs (the larger the circle, the greater the number of DEGs) and the color represents the corrected p-value (the redder the color, the smaller the corrected p-value). **(B)** Analysis of KEGG enrichment, with bubble plots displaying the top 20 most significant pathway enrichments. **(C)** Analysis of DO enrichment, with bubble plots displaying the top 20 most similar diseases. **(D)** Gene set enrichment analysis of GO showing the top five most significant biological processes. **(E)** Gene set enrichment analysis of KEGG, showing the top five most significant pathways.

In order to better understand the potential mechanisms of ERDEGs in OA, we performed GO, KEGG, and DO enrichment analyses of ERDEGs using the “clusterProfiler” package. GO enrichment analyses showed that the first five ERDEGs enrichments were mainly involved in regulation of peptidase activity, ameboidal−type cell migration, viral process, viral life cycle and regulation of endopeptidase activity. The top five ERDEGs enriched in cellular components and molecular functions are shown in **Figure 3A**. In addition, KEGG pathway analysis showed that these ERDEGs were enriched in the fluid shear stress and atherosclerosis, salmonella infection, regulation of actin cytoskeleton, human cytomegalovirus infection and pathogenic Escherichia coli infection, and that these pathways interacted closely with each other (**Figure 3B**). DO enrichment analysis revealed disease types with similar pathogenic mechanisms to ERDEGs in OA, such as atherosclerosis, arteriosclerotic cardiovascular disease, arteriosclerosis, breast carcinoma and familial hyperlipidemia (**Figure 3C**). **Supplementary Tables S5**–**7** show the detailed results of GO, KEGG and DO enrichment of ERDEGs.

### GSEA enrichment analysis

We investigated the enrichment of GO and KEGG pathways in OA by GSEA method and showed the five gene functions and pathways that were most significantly up-regulated in OA, respectively. GO enrichment results showed that cell chemotaxis, external encapsulating structure organization, collagen containing extracellular matrix, external encapsulating structure and extracellular matrix structural constituent were significantly upregulated (**Figure 3D**). KEGG pathway enrichment results showed that cytokine receptor interaction, ecm receptor interaction, focal adhesion, hematopoietic cell lineage and prion diseases were significantly upregulated (**Figure 3E**). Detailed results of the GSEA are shown in **Supplementary Tables S8**, **9**.

### Identification and validation of Hub OA-ERDEGs

**Figure 4:**
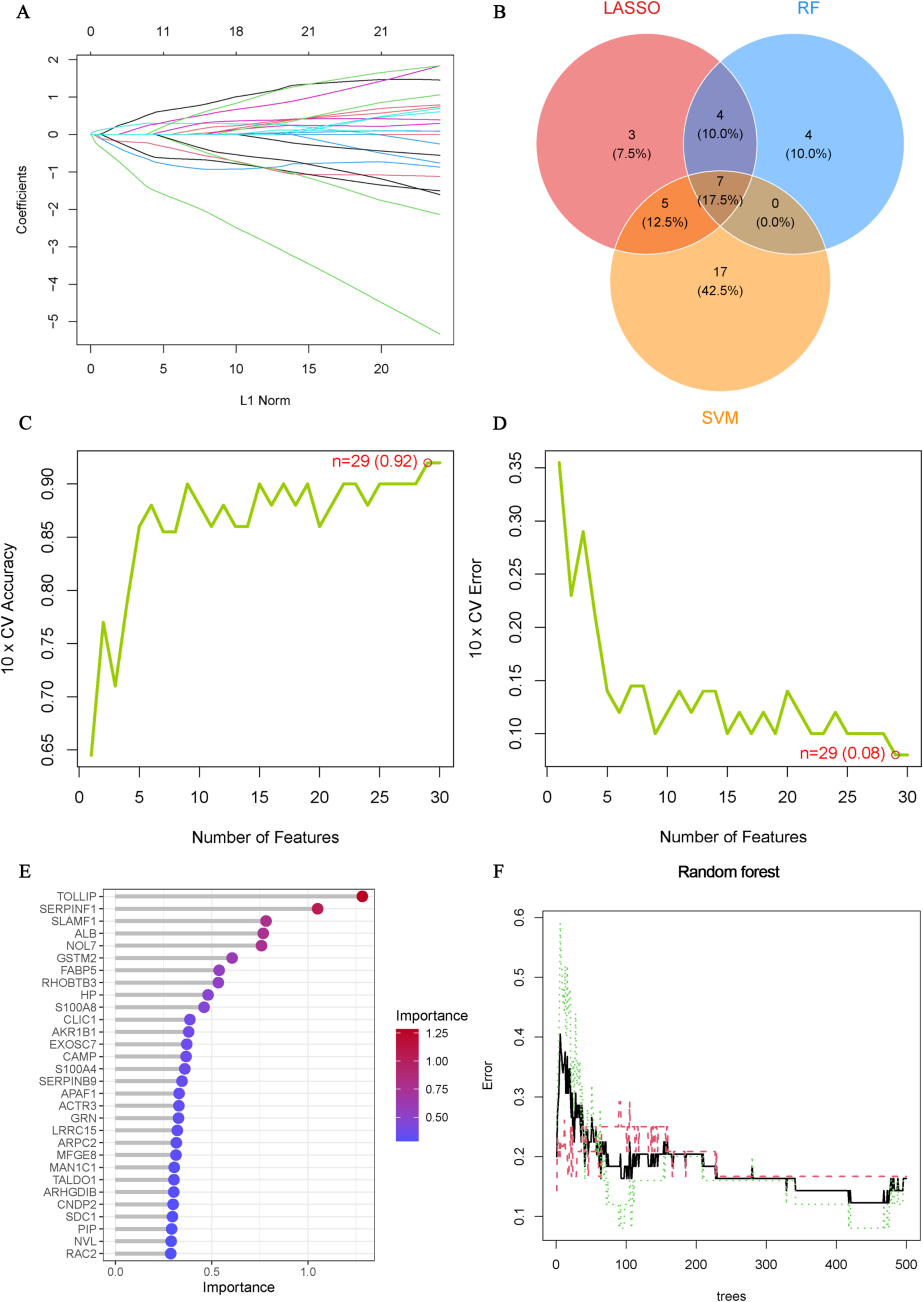
Machine Learning Screening Hub OA-ERDEGs. **(A)** Cross-validations of the choice of adjustment parameters in the LASSO model. Each curve corresponds to one gene. **(B)** LASSO, SVM-RFE and Random Forest algorithms for screening Venn diagrams of the OA-ERDEGs. **(C, D)** Maximum accuracy and minimum error plots of the SVM-RFE algorithm for screening optimal OA-ERDEGs. **(E)** Ranking of the relative importance of OA-ERDEGs. **(F)** Relationship between the number of random forest trees and the error rate.

**Figure 5:**
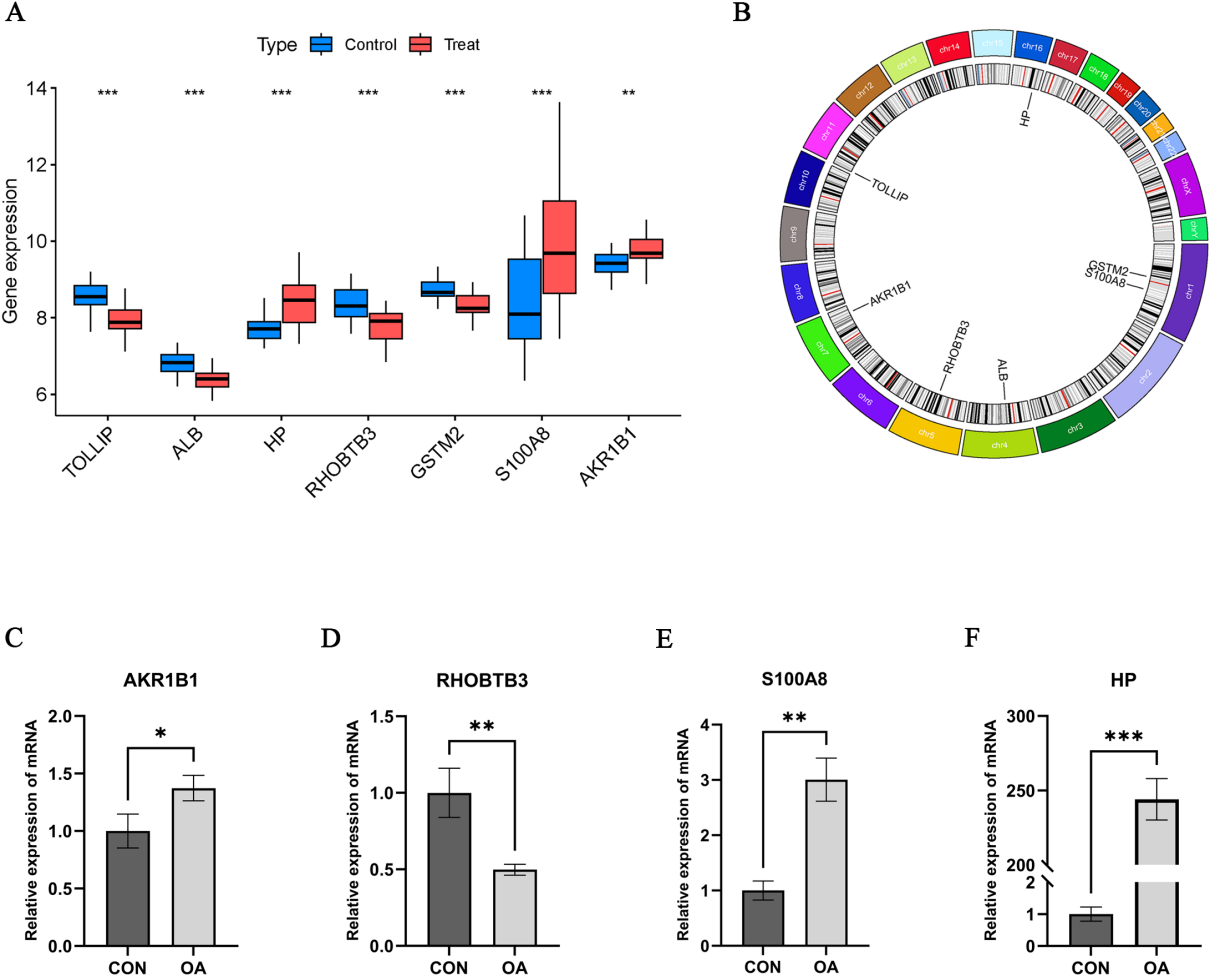
Hub OA-ERDEGs expression difference and validation. **(A)** Expression of 7 hub genes in OA samples. **(B)** Chromosome distribution of the 7 OA-ERDEGs. **(C-F)** The mRNA expression levels of Hub OA-ERDEGs were detected by qRT-PCR. Among them, AKR1B1, RHOBTB3, S100A8 and HP were significant. *p<0.05, **p<0.01, ***P<0.001.

To further understand the role of 105 ERDEGs in the diagnosis and prognosis of OA, we aimed to identify hub genes from these 105 ERDEGs to construct a diagnostic prediction model. We used three nonlinear machine learning algorithms, lasso (**Figure 4A**), SVM-RFE (**Figures 4C, D**), and Random Forest (**Figures 4E, F**) to screen OA-ERDEGs. The results of the three machine learning algorithms for recognizing Hub OA-ERDEGs are shown in **Supplementary Table S10**. Finally, by combining the results of the three algorithms, a total of nine Hub OA-ERDEGs were obtained, namely, TOLLIP, ALB, HP, RHOBTB3, GSTM2, S100A8 and AKR1B1 (**Figure 4B**). TOLLIP, ALB, RHOBTB3, and GSTM2 were down-regulated in OA samples and HP, GSTM2, and S100A8 were up-regulated in OA samples (**Figure 5A**). The location of each gene in the chromosome is shown in **Figure 5B**.

### qRT-PCR

We performed qRT-PCR assays to further validate the mRNA expression levels of Hub OA-ERDEGs. The results showed that four genes were expressed meaningfully, of which AKR1BI, S100A8 and HP were significantly upregulated in OA cartilage samples, and RHOBTB3 was significantly downregulated in cartilage samples (p-value less than 0.05), which was consistent with the expression in the training set (**Figures 5C–F**).

### Construction of Hub OA-ERDEGs risk prediction model

**Figure 6:**
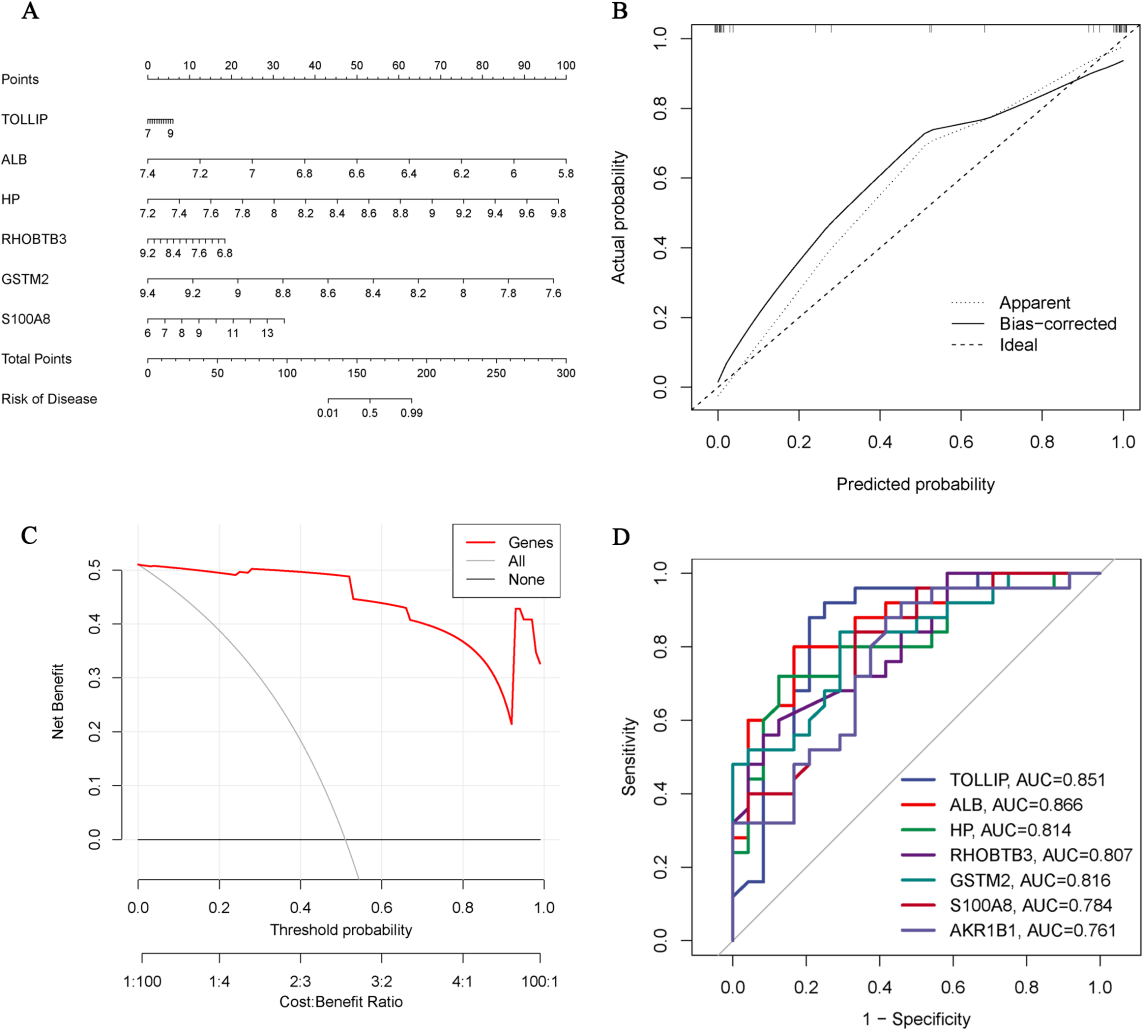
Hub OA-ERDEGs risk prediction model. **(A)** Nomogram of Hub OA-ERDEGs in the diagnosis of OA patients. **(B)** Calibration curve used to estimate the predictive accuracy of the nomogram (the closer to the ideal dashed line, the more reliable the result). **(C)** Accuracy of the clinical decision curve detection model (the further the red line endpoints are from the grey line, the higher the accuracy). **(D)** ROC curve analysis of Hub OA-ERDEGs and nomogram in the training set.

We constructed a nomogram of OA diagnosis based on the expression of Hub OA-ERDEGs to obtain a more applicable OA diagnosis model. By constructing the clinical calibration curves (**Figure 6A**) and clinical decision curves (**Figure 6B**) of the model, it is obvious that the model has a high predictive ability for OA. The score of each gene expressed in the column line graph accurately predicted the risk of osteoarthritis disease (**Figure 6C**).

### Diagnostic value of Hub OA-ARDEGs

ROC curve analysis showed that 7 Hub OAARDEGs and column line graphs had high diagnostic value for OA.ALB and column line graphs had the highest diagnostic value (AUC=0.866), and the diagnostic values of other genes were: TOLLIP (AUC=0.851), GSTM2 (AUC=0.816), and HP (AUC=0.814), RHOBTB3 (AUC=0.807), S100A8 (AUC=0.784) and AKR1B1 (AUC=0.761) (**Figure 6D**). Therefore, the seven Hub OA-ERDGEs can be used as reliable biomarkers with high diagnostic value for the diagnosis of OA.

### Immune infiltration analysis

**Figure 7:**
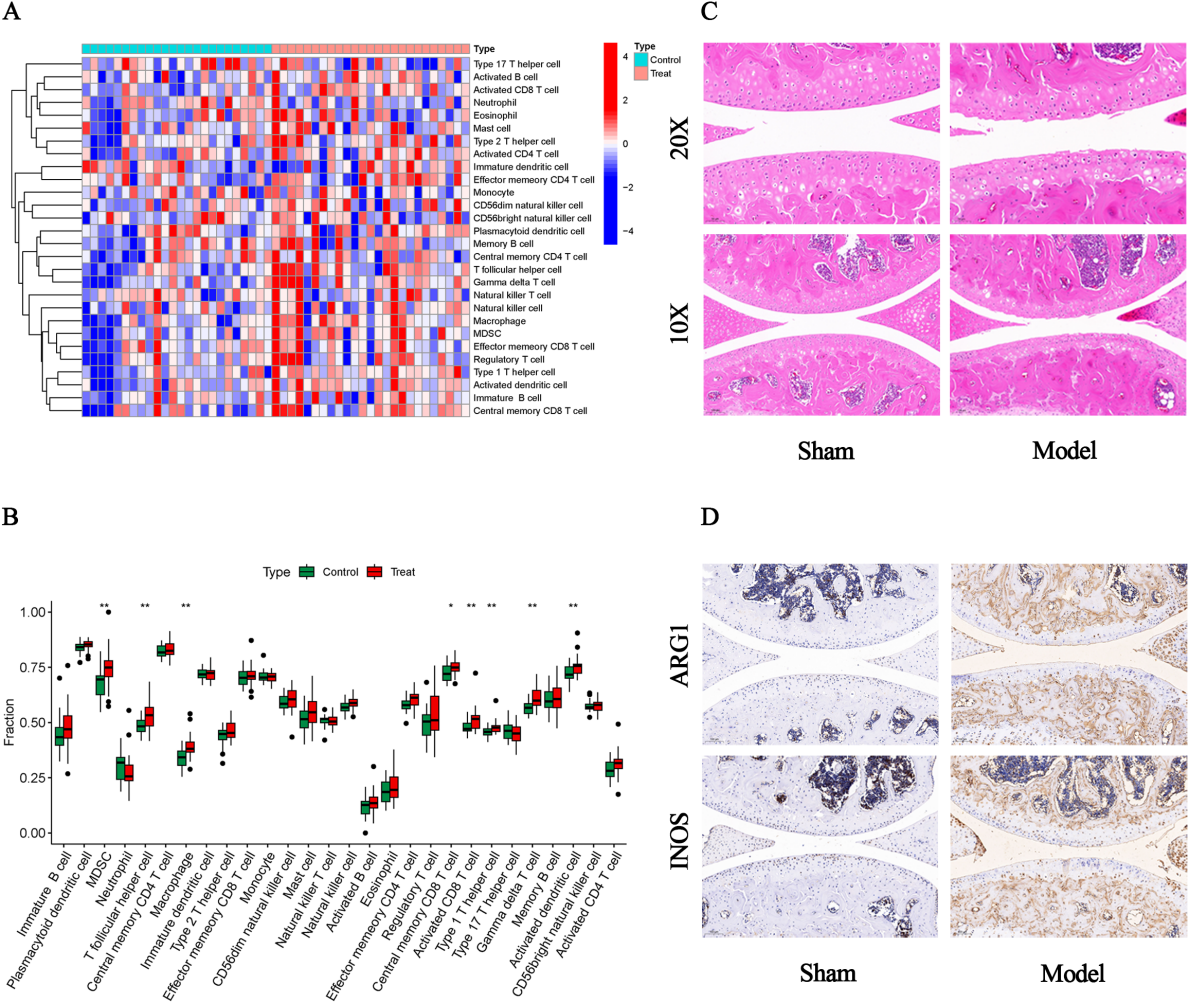
Immune infiltration and histological verification. **(A)** Heat map of the differences in the distribution of 28 immune cells in each sample. **(B)** Box plots of differences in the infiltration of 28 immune cells. **(C)** Representative HE staining images (10x and 20x). **(D)** Representative immunohistochemistry images of INOS and ARG1 (10x).

**Figure 8:**
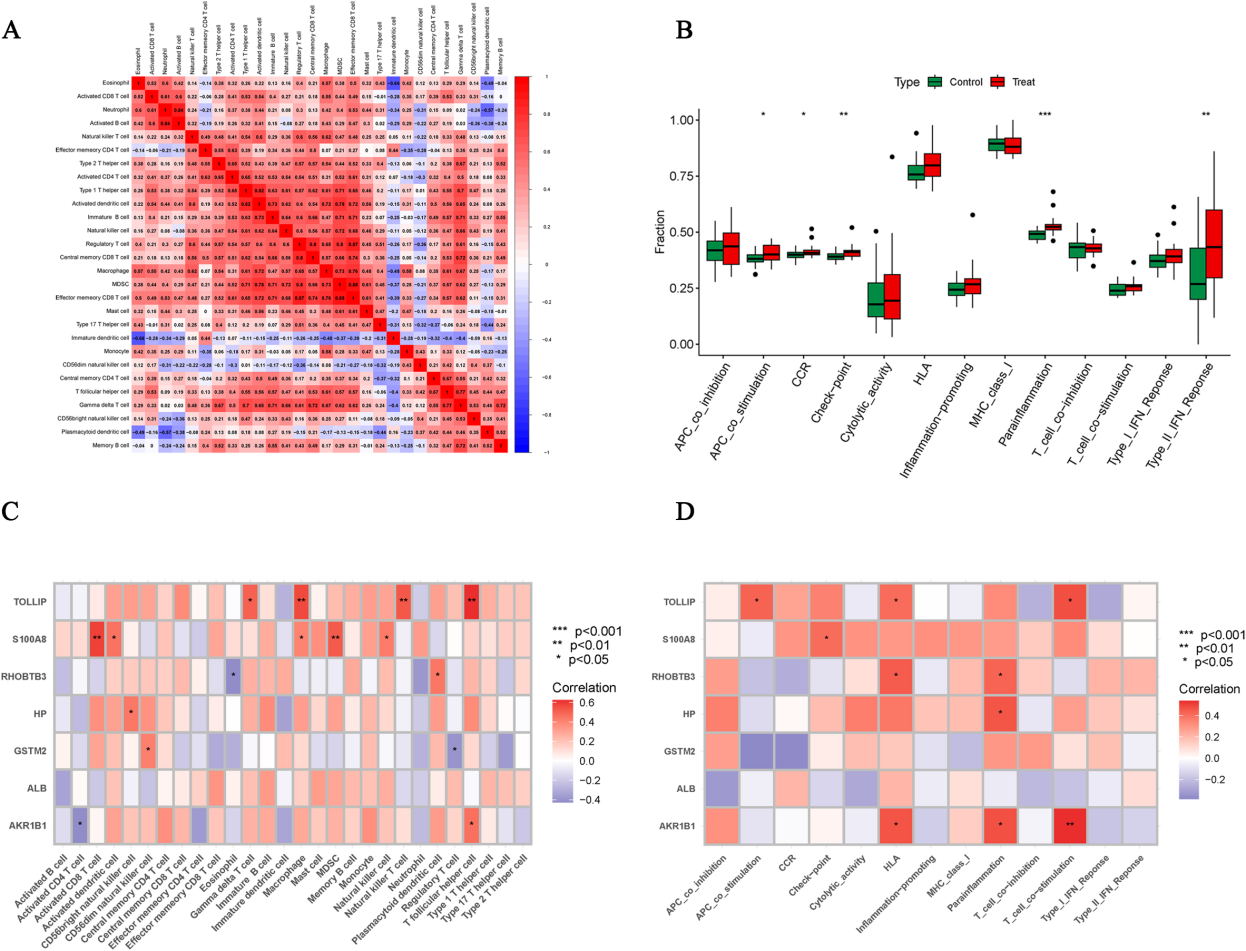
Correlation analysis of immune cells and immune function. **(A)** Correlation analysis between 28 immune cell species. **(B)** Box plots of differences in the 13 immune functions in normal cartilage and OA cartilage sample. **(C, D)** Correlation analysis of Hub OA-ERDEGs with 28 immune cells and 13 immune functions. *P<0.05, **P<0.01, ***P<0.001.

We used the ssGSEA algorithm to find myeloid-derived suppressor cells, T follicular helper cell, Macrophage, central memory CD8 T cell, activated CD8 T cell, type 1 T helper cell, gamma delta T cell and activated dendritic cell infiltration were significantly increased (**Figures 7A, B**). Subsequently, to investigate histological infiltration of immune cells, an OA animal model was established through DMM surgery. During OA progression, characteristic pathological manifestations including cartilage degradation and subchondral bone sclerosis were observed (**Figure 7C**). Furthermore, upregulated expression of immune cell markers such as INOS and ARG1 was detected in joint tissues of OA group mice (**Figure 7D**). Spearman correlation was used to analyze the interactions between immune cells. The results showed significant correlations between most immune cells, for example, effector memory CD8 T cell was significantly positively correlated with MDSC (r=0.88) (**Figure 8A**). Para inflammation, check−point, type II IFN response and other immune functions were significantly activated in OA samples (**Figure 8B**). AKR1B1, TOLLIP, RHOBTB3, HP and S100A8 were well correlated with multiple immune functions (**Figures 8C, D**). Data of results were shown in the **Supplementary Tables S11**, **12**.

## Discussion

In recent years, the role of exosomes in OA, as an important mediator of intercellular communication, has gradually gained attention in research. Recent studies have shown that exosomes not only carry a variety of bioactive molecules, but also participate in key signaling pathways in the pathological process of OA (29). For example, microRNAs enriched in exosomes (e.g., miR-146a, miR-140, etc.) have been shown to regulate signaling pathways associated with inflammation and cartilage degradation. These microRNAs are involved in the regulation of classical pathways such as NF-κB, Wnt/β-catenin, etc. by affecting the expression of target genes, which contributes to the pathologic process of OA (30). In addition, it has been shown that exosome-derived proteins also play an important role in the development of OA. Proteins in exosomes are not only associated with intercellular signaling, but may also affect the proliferation and differentiation of fibroblasts and chondrocytes, which in turn affects the metabolic homeostasis of articular cartilage (31). Notably, the interaction between exosomes and immune cells is also one of the research focuses. Through the release of inflammatory factors and cytokines from exosomes, the activity and infiltration of immune cells can be modulated to influence the inflammatory response in OA. This process may influence the clinical manifestations and course of OA by regulating the accumulation and release of exosomes in the joint cavity (32). Therefore, exploring in depth the role of exosomes and related signaling pathways in OA not only provides new perspectives for understanding the pathogenesis of the disease, but also lays the foundation for seeking new biomarkers and therapeutic targets in the clinic.

In normal and OA cartilage samples, we identified 105 ERDEGs. and further screened by machine learning, 7 Hub OA-ERDEGs (TOLLIP, ALB, HP, RHOBTB3, GSTM2, S100A8, AKR1B1) were identified. Our findings suggest that Hub OA-ERDEGs have excellent diagnostic ability to predict OA and are significantly up- or down-regulated in cartilage samples from OA patients. TOLLIP is considered a negative regulator of the Toll-like receptor (TLRs) signaling pathway. It inhibits the NF-κB and MAPK signaling pathways by binding to TLRs, further inhibiting the activation of downstream signals, thereby reducing the intensity of the inflammatory response (33). The ALB gene encodes albumin, a plasma protein synthesized primarily by the liver that has a variety of physiological functions, and recent studies have found that the ALB gene and its products play an important role in the inflammatory response. For example, albumin binds and transports antioxidants and reduces oxidative stress, thereby decreasing the inflammatory response. In addition, albumin is able to regulate cytokine secretion and reduce inflammation through interactions with immune cells (34). HP is primarily responsible for encoding acute phase protein-hemoglobin binding proteins in the organism. During tissue injury or inflammation, erythrocyte rupture releases hemoglobin, and free hemoglobin has strong oxidative properties that may lead to cellular damage. HP reduces oxidative stress by binding to free hemoglobin, forming a complex that facilitates its clearance by macrophages (35). RHOBTB3 gene is a gene encoding a member of the Rho GTPase family, and it has been pointed out that the expression level of RHOBTB3 is significantly increased in breast cancer tissues. And it can promote breast cancer progression by regulating the expression of Collagen Type I Alpha 1 Chain (COL1A1) (36). Its high expression in OA samples was also found in this study, so we hypothesized that RHOBTB3 is also involved in inflammation in osteoarthritis. The GSTM2 gene is a member of the glutathione S-transferase family, which maintains the MAPK signaling pathway by directly interacting with apoptosis signal-regulated kinase 1 (ASK1) to scavenge free radicals and oxidative products *in vivo*, thereby reducing the level of oxidative stress and indirectly decreasing the degree of inflammatory response (37). The enzyme encoded by the AKR1B1 gene is mainly responsible for reducing a variety of aldehydes to their corresponding alcohols, and studies have indicated that Linarin has anti-inflammatory, antioxidant, and anti-apoptotic effects in osteoarthritis, and Linarin reduces the intensity of the inflammatory response by inhibiting endoplasmic reticulum stress and further inhibiting AKR1B1 (38). The S100A8 gene encodes a small protein belonging to the S100 family of proteins, which play important roles in many biological processes, including cell proliferation, differentiation, inflammation, and the immune response. The S100A8 protein usually associates with the S100A9 protein to form a dimer, the S100A8/S100A9 complex, which promotes the aggregation of monocytes and other inflammatory cells, thereby exacerbating the inflammatory response and tissue damage (39). It has been noted that the mRNA expression levels of S100A8 and S100A9 are significantly increased in the early stages of OA, but their expression in cartilage gradually decreases as the disease progresses. This suggests that these two proteins may promote cartilage degradation by up-regulating matrix metalloproteinases (MMPs) and aggrecans in the early stages of OA, while this sustained effect is lost in the later stages of OA. In contrast, in inflammatory arthritis, S100A8 and S100A9 show more sustained expression and activity (40). S100A8 was significantly up-regulated in OA samples in the present study, which may indicate that the OA model resulting from stimulation of chondrocytes with IL-1β for 24h is at an early stage of the disease, highly consistent with the results of previous studies.

Inflammatory responses in osteoarthritis exacerbate damage to articular cartilage and synovium through the release of pro-inflammatory factors and mediators, leading to joint pain and dysfunction; while immune deficiencies may deregulate these inflammatory processes, further exacerbating inflammatory processes and degenerative joint disease (41). T follicular helper (TFh) cells are a specific class of CD4^+^ T cells that are primarily responsible for promoting B cell maturation and antibody production in the germinal centers of the lymph nodes. TFh cells may be involved in the pathological process of arthritis through the production of cytokines, such as IL-21, and the modulation of B cell activity (42). It has been suggested that the expression of long chain non-coding RNA (Lnc RNA) MM2P in monocyte-derived cells promotes the polarization of M2-type macrophages and the delivery of Sox9 mRNA and protein via their secreted exosomes, thereby enhancing the differentiation and function of primary chondrocytes (43). Dendritic cells are important antigen-presenting cells that play a key role in the immune response. Exosomes produced by dendritic cells can deliver mRNAs, miRNAs, and cytokines and interact with immune cells (44). Myeloid-derived suppressor cells (MDSCs) are a class of cells with immunosuppressive functions, mainly derived from bone marrow, capable of suppressing the activity of T cells and other immune cells through multiple mechanisms. In the tumor microenvironment, MDSCs promote tumor cell growth and metastasis as well as suppress anti-tumor immune responses by secreting exosomes (45). We hypothesized that in some inflammation-related diseases, such as osteoarthritis, exosomes produced by MDSCs could similarly play an immunomodulatory role. We used Spearman correlation analysis to show that nine Hub OA-ERDEGs were reasonably correlated with immune cell function, suggesting that these genes influence immune cell function and play a key role in the development and progression of OA.

This study has several limitations. First, the intersection of non-chondrocyte-derived exosomal genes with OA chondrocyte datasets may result in gene omission due to the absence of cell type-specific exosomal gene references. While data screening and processing enhanced the reliability of candidate genes, future validation through chondrocyte-specific exosome isolation combined with multi-omics analysis remains necessary. Second, algorithm-dependent variability (LASSO/SVM/RF) may affect result stability, though our multi-model integration aligns with current multi-omics standards. Third, the sample size (24 controls/25 OA cases across four datasets), though geographically diverse, remains modest, necessitating multicenter validation. Finally, while qRT-PCR confirmed expression trends of AKR1B1, RHOBTB3, S100A8, and HP in OA chondrocytes, their diagnostic specificity, functional mechanisms, and clinical utility require further exploration through large-scale cohorts, *in vitro*/*in vivo* models, and differential diagnosis assays. These limitations highlight the need for expanded validation frameworks to advance the translational potential of our findings.

### Ethics

All summary statistical data used for screening Hub genes were generated from previous studies, and all original studies obtained ethical approval and personal consent.

## Conclusion

In summary, this study preliminarily explored the potential linkage of differential and exosome-related genes in OA cartilage tissues. In addition, seven Hub OA-ERDEGs have excellent diagnostic ability for OA, these genes may become new targets for OA diagnosis and treatment. However, more experimental studies are needed to support our findings.

## Data Availability

The original contributions presented in the study are included in the article/**Supplementary Material**. Further inquiries can be directed to the corresponding authors.
